# The Efficacy, Safety, and Tolerability of Probiotics on Depression: Clinical Results From an Open-Label Pilot Study

**DOI:** 10.3389/fpsyt.2021.618279

**Published:** 2021-02-15

**Authors:** Caroline J. K. Wallace, Roumen V. Milev

**Affiliations:** ^1^Centre for Neuroscience Studies, Queen's University, Kingston, ON, Canada; ^2^Department of Psychiatry, Queen's University, Kingston, ON, Canada

**Keywords:** depression, probiotics, anxiety, gut-brain axis, pilot study

## Abstract

**Background:** A growing body of research has shown that consumption of probiotics can improve symptoms associated with mood and anxiety disorders through activity of the gut-brain axis. However, the effects of probiotics have yet to be tested in a clinical sample of treatment-naïve patients diagnosed with Major Depressive Disorder (MDD). The aim of this 8-week, open-label pilot study is to examine changes in depressive symptoms before and after the introduction of a probiotic supplement in 10 treatment-naïve MDD patients and to provide data on the feasibility of conducting a larger double-blind, randomized, placebo-controlled trial in the same patient population. Here we report on the clinical outcome measures of the study.

**Methods:** Participants recruited from the community in Kingston, Ontario, Canada consumed a probiotic supplement containing *Lactobacillus helveticus* R0052 and *Bifidobacterium longum* R0175 (CEREBIOME^®^) at a dose of 3 × 10^9^ CFU once per day for 8 weeks. Clinical symptoms of depression were measured using a validated battery of clinical scales and self-report questionnaires (CAN-BIND protocol). Data was collected at baseline, week 4, and week 8.

**Results:** Significant improvements in affective clinical symptoms were observed at week 4 and were sustained at week 8. Significant improvements in subjective sleep quality were observed by week 8. No side effects or adverse effects associated with the probiotic supplement were observed.

**Conclusions:** The findings from this study support the existing evidence in this emerging field for probiotics having a role in alleviating symptoms of depression in treatment-naïve, moderately depressed patients and indicate that the probiotic supplement is safe and well-tolerated in this population. However, further comprehensive studies are required to draw conclusions.

## Introduction

Recent advances in gut-brain axis research have linked psychiatric disorders such as depression to changes in the microbiome of the gastrointestinal (GI) tract (1). Several experimental methods have been used to examine the microbiota-gut-brain axis and the effects that these bacteria in the GI tract may have on central nervous system functioning, including antibiotic treatment, germ-free animal models, and fecal microbiota transplantation. With respect to psychiatric disorders, one emerging approach that has been demonstrated by both preclinical and preliminary clinical research to have a potential therapeutic role is probiotic supplementation (2). Probiotics are live bacteria that colonize the GI tract and exert a health benefit on the host (3). Probiotics can be obtained through consumption of naturally fermented foods, functional foods that are fortified in such bacteria, or in supplement form.

A wealth of preclinical data has shown that probiotic consumption improved depression- and anxiety-like behavior in rodent models (4–6) and have posited inflammatory activity through the gut-brain axis as driving these effects. However, whether probiotic consumption positively changes microbiome composition and function in humans to result in improved mood and reduced depressive symptoms remains unclear. Several randomized control trials (RCTs) have explored this possibility in clinically depressed patients (7–12), but inconsistencies in sample characteristics, bacterial species and strains, dosage, and duration of intervention resulted in mixed findings. There also remain significant gaps in the research, such as probiotics' effects on sleep quality, which is often disrupted in depression and leads to poor functional outcomes. Further, most studies have examined probiotics in patients who were also taking antidepressant medication. While exploring probiotics in combination with standard antidepressant medication is important to see whether they can be effective as an adjunctive treatment, it is crucial that we also examine probiotics as a standalone treatment to determine whether there are any pure effects and if they can be translated into an effective monotherapy. This research comes at a time when patients and healthcare professionals are looking for more personalized, tolerable, and effective ways to alleviate symptoms of depression, and as scientists are discovering further evidence that the effects of bacterial activity in the gut is not confined to the gastrointestinal system.

To begin to address in the gaps in the research, we conducted an open-label pilot study to assess the effects of probiotics on Major Depressive Disorder (MDD) in a treatment-naïve (i.e., have no prior history of antidepressant medication use) clinical sample. The objective of this study was to examine the safety, tolerability, and efficacy of a probiotic supplement on symptoms of depression in treatment-naïve depressed patients. The ultimate goal of the study was to provide data on the feasibility of conducting a larger double-blind, randomized, placebo-controlled trial in the same patient population. Here we report on the clinical outcome measures of the study. Secondary outcome measures, which include levels of circulating inflammatory and gut permeability markers, microbiome composition, and sleep architecture, involved the collection of blood and stool samples and polysomnography and will be reported on when data becomes available.

## Materials and Methods

### Study Design

This study was an 8-week open-label exploratory study. All participants were unblinded and received the active investigational product for 8 weeks. The study was registered with ClinicalTrials.gov in 2016 (NCT02838043) and obtained ethics clearance from the Queen's University Health Sciences and Affiliated Teaching Hospitals Research Ethics Board (protocol number 6016312). Authorization from Health Canada was obtained for use of a probiotic supplement containing *Lactobacillus helveticus* R0052 and *Bifidobacterium longum* R0175, registered with the Natural and Non-Prescription Directorate of Health Canada.

### Participants

The target participant population was males and females ages 18–65 in a current episode of MDD as determined by the Mini International Neuropsychiatric Interview (MINI) (13) per DSM-IV criteria, and not currently taking any antidepressant medication. Full inclusion and exclusion criteria can be found in Table 1. Participants who were undergoing behavioral and/or cognitive therapy for depression were eligible for the trial if they had been undergoing the therapy for at least 6 months and would continue at their regular frequency during the trial for consistency. Participants were recruited from the community via paper and web-based advertisements. Interested potential participants contacted study personnel by email and were followed up with by a pre-screening phone call. If eligible, based on the pre-screening criteria, potential participants then underwent a full screening visit at the study site.

**Table 1:** Full inclusion and exclusion criteria.

**Inclusion criteria**	**Exclusion criteria**
1. Diagnosis of MDD by MINI 2. Current depressive episode with a MADRS score of ≥ 20 3. Males and females between ages 18 and 65 4. Able to understand and comply with the requirements of the study 5. Provision of written informed consent	1. Use of any antidepressant drug 2. Use of any antibiotic drug in the past 4 weeks 3. Use of any sleep medication in the past 4 weeks 4. Milk, yeast, or soy allergy 5. History of alcohol or substance abuse in the past 6 months 6. Daily use of probiotic product in the past 2 weeks 7. Use of any type of laxative 8. Consumption of products fortified in probiotics 9. Severely suicidal 10. Experiencing psychosis or bipolar episode 11. History of epilepsy or uncontrolled seizures 12. Women who are pregnant, breastfeeding, or planning to become pregnant during the trial 13. Immunodeficiency 14. Unstable medical conditions or serious diseases/conditions (e.g., cancer, cardiovascular, renal, lung, diabetes, psychiatric illness, bleeding disorders, etc.) 15. Use of natural health products (NHPs) that affect depression (e.g., St. John's Wort, passionflower, etc.) 16. Electroconvulsive therapy (ECT) in the year prior to participation in the study 17. Taking antidepressant medication or other not-permitted treatment that cannot be safely discontinued

### Setting

Clinical data collection was carried out in a tertiary-care mental health and continuing care hospital in Kingston, Ontario, Canada.

### Investigational Product

The supplement under investigation is a probiotic formulation containing two active ingredients: *Lactobacillus helveticus* R0052 (90%) and *Bifidobacterium longum* R0175 (10%) (CEREBIOME^®^, Lallemand Health Solutions Inc., Mirabel, Canada). The probiotic supplement is packaged in 1.5 g sachets as a lyophilized powder at a dose of 3 × 10^9^ colony forming units (CFU) per sachet and is micro-encapsulated to prevent breakdown by stomach acid, ensuring the bacteria reaches the GI tract. The supplement also contains the following excipients: xylitol (sweetener), maltodextrin (coating agent), fruit flavor and malic acid (acidity regulator), yeast extract, sucrose, and ascorbic acid (cryoprotectants). Participants were instructed to refrigerate the investigational product and consume once daily at any time of day. If a dose was missed, participants were instructed to skip the missed dose and continue with the probiotic the following day.

### Measures

#### Clinical

Clinical symptoms of depression were measured using a battery of validated clinical scales and self-report questionnaires from the Canadian Biomarker Integration Network in Depression (CAN-BIND) studies. The primary outcome measure was overall depressive symptoms, measured using the clinician-rated Montgomery-Åsberg Depression Rating Scale (MADRS) (14). For inclusion in the study, participants must have had a baseline MADRS score of ≥20, indicating at least a moderate depression. Depressive symptoms were also measured using the Quick Inventory of Depressive Symptomatology (QIDS-SR16) (15), a sixteen-item self-report questionnaire. Anhedonia was measured with the Snaith-Hamilton Pleasure Scale (SHAPS) (16), which assesses one's ability to experience pleasure or enjoyment, and anxiety with the Generalized Anxiety Disorder 7-item scale (GAD-7) (17) and the State Trait Anxiety Inventory (STAI) (18). Sleep was measured subjectively using the Pittsburgh Sleep Quality Index (PSQI) (19). Basic information on diet was collected using a brief diet questionnaire, and adverse gastrointestinal symptoms were recorded with a gastrointestinal symptom rating scale. Clinical data was collected during visits to the study site at baseline, week 4, and week 8.

### Analyses

#### Statistical Analyses

A series of one-way repeated measures analyses of variance (ANOVAs) and *post-hoc* tests using Bonferroni correction were conducted to examine changes in clinical measures from baseline to week 4 and week 8. Data was analyzed using two-tailed techniques with IBM's statistical program Statistical Package for the Social Sciences (SPSS) version 24 with a significance level of α = 0.05.

## Results

### Sample Characteristics

All 10 participants completed the study and were included in clinical data analysis reported here. The sample was 70% female, with an age range of 19–41 years (average age: 25.2 ± 7 years). Baseline MADRS scores ranged from 20 to 31 with a mean of 24.9, indicating all participants were experiencing a moderate depression severity. All baseline (week 0) demographic information can be found in Table 2. Importantly, no side effects or adverse events associated with the probiotic supplement were noted.

**Table 2:** Summary of study sample demographics.

**Demographics**	
Total participants (n)	10
Female	7
Male	3
**Age (years)**	
Mean ± SD	25.2 ± 7
Range	18–41
**Symptom severity at baseline (MADRS score)**	
Mean ± SD	24.9 ± 3.4
Range	20–31

### Clinical Results

**Figure 1:**
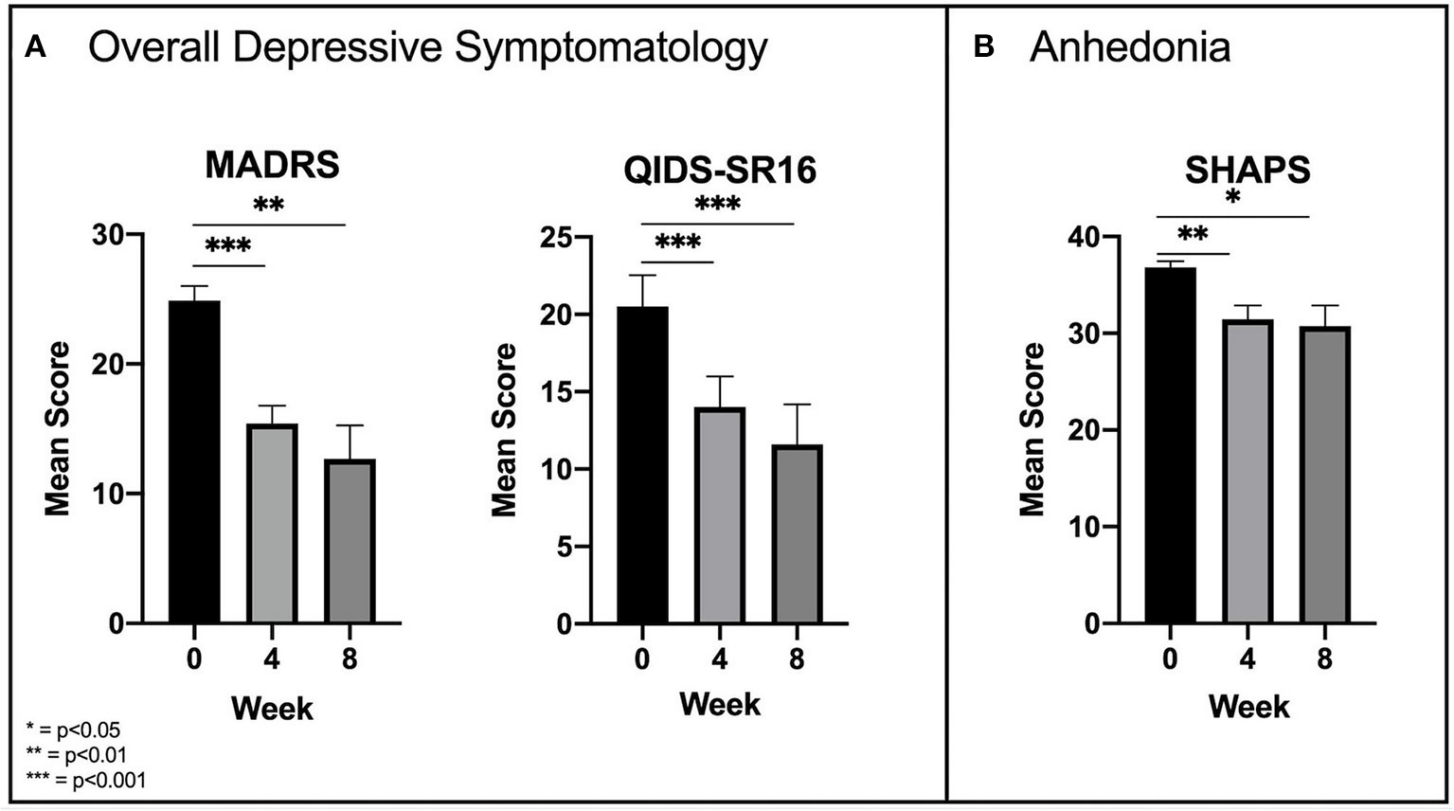
**(A)** Early significant improvements in overall depressive symptomatology as measured by the MADRS and QIDS-SR16. **(B)** Similar significant improvements in anhedonia as measured by the SHAPS.

Overall depressive symptomatology was measured using the MADRS and the QIDS-SR16. Analyses found significant reductions in mean MADRS scores between time points [*F*_(2, 18)_ = 26.84, *p* < 0.001]. Pairwise comparisons revealed a significant (*p* < 0.001) reduction from baseline (24.9 ± 3.4) to week 4 (15.4 ± 4.4), but only a slight reduction from week 4 to week 8 (12.7 ± 8.1) which was not significant (*p* = 0.377) (Figure 1A). Similarly, there were significant reductions in mean QIDS-SR16 scores [*F*_(2, 18)_ = 38.45, *p* < 0.001] with significant reductions from baseline (20.5 ± 6.4) to week 4 (14 ± 6.3) and only a slight reduction from week 4 to week 8 (11.6 ± 8.1) which was not significant (*p* = 0.126) (Figure 1A). Analysis of anhedonia, measured with the SHAPS, found significant reductions in mean SHAPS scores [*F*_(2, 18)_ = 9.97, *p* = 0.001] and like overall depressive symptoms, an initial significant (*p* = 0.004) reduction from baseline (36.8 ± 2) to week 4 (31.4 ± 4.6) followed by a slight non-significant (*p* = 1.000) reduction from week 4 to week 8 (30.7 ± 6.8) (Figure 1B).

**Figure 2:**
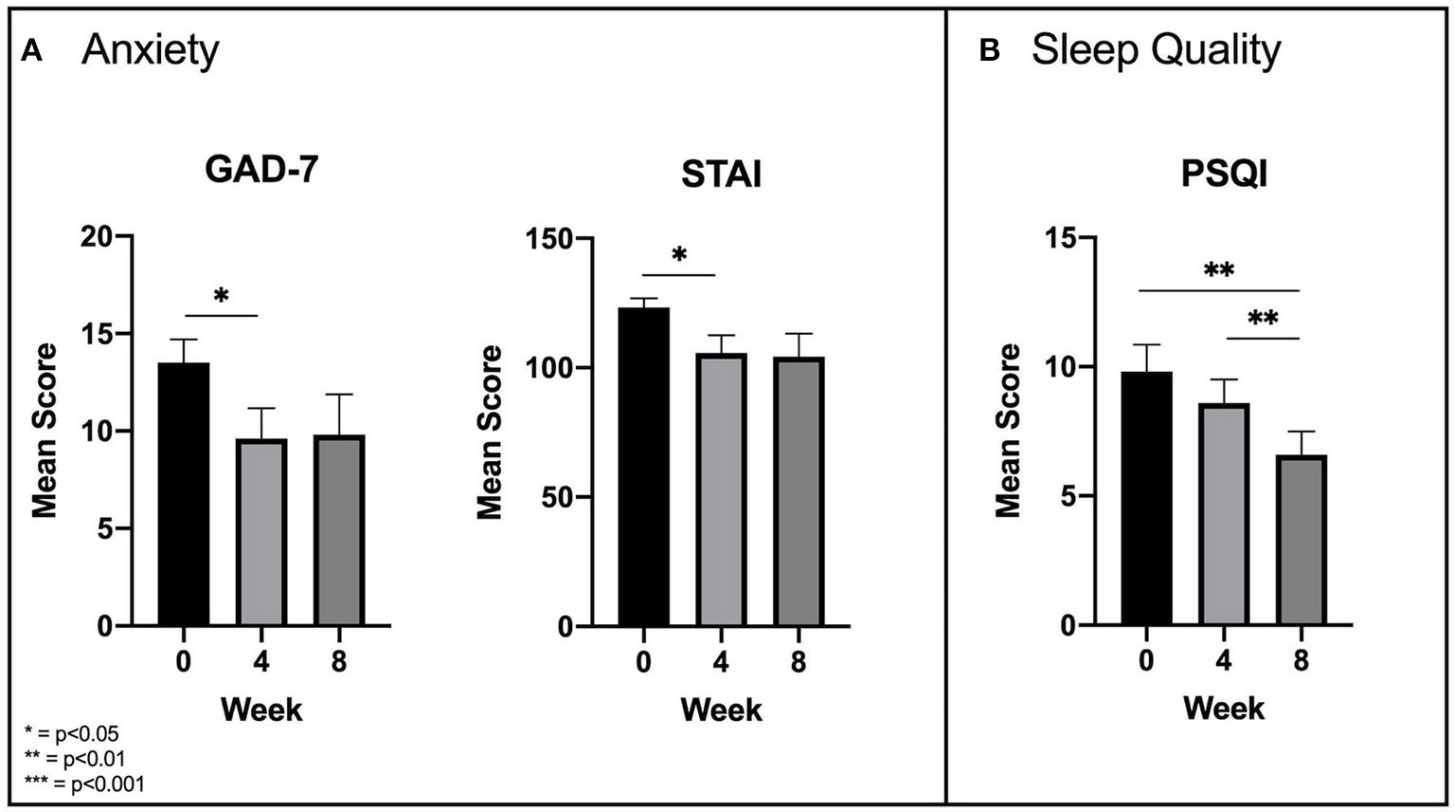
**(A)** Significant improvements in anxiety as measured by the GAD-7 and STAI. **(B)** Delayed significant improvements in subjective sleep quality as measured by the PSQI.

Anxiety was measured using the GAD-7 and the STAI. An ANOVA with a Greenhouse-Geisser correction determined that there were significant [*F*_(1.25, 11.28)_ = 5.58, *p* = 0.031] reductions in GAD-7 scores across time points, but only from baseline to week 4 (13.5 ± 3.8 and 9.6 ± 4.9, respectively; *p* = 0.021) and not from week 4 to week 8 (9.8 ± 6.6; *p* = 1.000) (Figure 2A). An ANOVA with sphericity assumed conducted on mean STAI scores mirrored these results, finding a significant reduction [*F*_(2, 18)_ = 6.44, *p* = 0.008] in mean scores from baseline to week 4 (123.3 ± 11.3 and 105.7 ± 21.5, respectively; *p* = 0.016), but not from week 4 to week 8 (104.3 ± 28.1; *p* = 1.000) (Figure 2A).

Subjective sleep quality was measured with the PSQI. An ANOVA with a Greenhouse-Geisser correction found significant reductions in mean PSQI scores [*F*_(1.3, 11.6)_ = 10.76, *p* = 0.005]. Unlike the other clinical measures, there was no significant reduction (*p* = 0.167) in scores from baseline (9.8 ± 3.6) to week 4 (8.6 ± 2.9), but a significant reduction (*p* = 0.002) did emerge from week 4 to week 8 (6.6 ± 2.8) (Figure 2B).

## Discussion

While only preliminary, the findings from this study suggest a role for probiotics in alleviating depressive symptoms in moderately clinically depressed, treatment-naïve patients. Daily supplementation with probiotics significantly improved overall mood and anhedonia, reduced anxiety, and improved sleep quality. Moreover, the absence of side effects and adverse events associated with the probiotic indicates that it was well-tolerated and safe to use in this population. Our primary outcome measure, the MADRS, measures severity of overall depression symptoms. MADRS scores range from 0 to 60 with 0 to 6 indicating an absence of symptoms, 7 to 19 indicating a mild depression, 20 to 34 indicating a moderate depression, and >34 indicating a severe depression. In the current study, the significant reduction of mean MADRS scores from 24.9 at baseline to 15.5 at week 4 and 12.7 at week 8 suggests that while probiotic supplementation did not completely alleviate depressive symptoms, it improved symptom severity from moderate to mild.

While affective symptoms such as mood and anhedonia were improved by week 4, subjective sleep quality did not improve until week 8. This suggests that probiotics may exert their effects early on for affective symptoms, and after prolonged use for sleep symptoms. The reason for this is unclear, although a study examining the effects of a multistrain probiotic formulation on subjective sleep quality in healthy controls also did not report significant improvements until later (week 6) (20). A recent meta-analysis also supports this evidence, suggesting that improvements in subjective sleep quality using probiotics requires a treatment duration of 8 weeks or longer (21). It is possible that in our study, subjective sleep quality was indirectly improved by consumption of the probiotic, with improved affective symptoms acting as a moderator. The mean score of 9.8 on the PSQI at baseline indicates that participant's subjective sleep quality at the start of the study was poor. Given that a global score of 5 or more on the PSQI indicates poor sleep quality, the improvement to 6.6 at week 8 still indicates a poor sleep quality, However, the higher the score, the worse the quality.

To our knowledge, there have been eight previous trials (7–12, 22, 23) assessing the effects of a probiotic supplement on symptoms of depression in a depressed sample, mostly as adjuvant to pharmacological antidepressants. Six of these trials, discussed below, were RCTs, with two using the same combination of *L. helveticus R0052* and *B. longum R0175* as the present study. This is important as probiotics' effects are both species- and strain-specific. In 2016, Akkasheh et al. examined the effects of a probiotic capsule containing *L. acidophilus* (2 × 10^9^ CFU), *L. casei* (2 × 10^9^ CFU), and *B. longum* (2 × 10^9^ CFU) on symptoms of depression compared to a placebo in 40 Iranian patients diagnosed with MDD per DSM-IV criteria. After 8 weeks, the authors found a significant reduction in depression scores measured by the Beck Depression Inventory (BDI) (7), which has been shown to be highly correlated with the MADRS (24) and is consistent with the findings from the present study. In 2017, Romijn et al. conducted a double-blind, randomized, placebo-controlled trial in 79 Australian individuals presenting with low mood and included more outcome measures of depressive symptoms. This study used the same probiotic supplement as the present study, however, contrary to our findings, did not find any significant improvements on the MADRS, QIDS-SR16, Clinical Global Impressions scale (CGI), Global Assessment of Functioning (GAF), or Depression, Anxiety, and Stress Scale (DASS-42) (8) after 8 weeks. This may be because the authors did not exclude psychotherapy use or past antidepressant use, leading to a sample of potentially chronic and treatment-resistant depression, which is not the sub-sample of depressed patients we hypothesize a probiotic supplement will benefit. It also should be noted that Romijn et al. sample consisted of participants self-identified as being depressed and were not diagnosed by a clinician. In 2018, Ghorbani et al. from Iran conducted a 6-week blinded, placebo-controlled, randomized trial examining the effects of a synbiotic containing *L. casaei, L. acidophilus, L. bulgaricus, L. rhamnosus, B. breve, B. longum, Streptococcus thermophilus*, and fructooligosaccharide as the prebiotic. The synbiotic was examined in 40 patients diagnosed with MDD as an adjuvant to fluoxetine. Results showed that the synbiotic group had significantly lower depression scores on the Hamilton Depression Rating Scale (HAM-D) compared to the placebo (9). Also in 2018, Kazemi et al. conducted a double-blinded, randomized, placebo-controlled trial comparing the effects of the same probiotic supplement used in the present study as well as a prebiotic to a placebo. This study, also conducted in Iran, examined the probiotic and prebiotic as an adjunctive therapy to sertraline, fluoxetine, citalopram, or amitriptyline in 81 patients diagnosed with MDD. In line with Akkasheh et al. findings, pairwise comparisons revealed significant improvements in depression symptoms measured by the BDI in the probiotic group, but not in the prebiotic group (10). A 2019 study from Poland by Rudzki et al. examined the effects of *L. plantarum 299v* as an adjuvant to selective serotonin reuptake inhibitors (SSRIs) in a double-blind, randomized, placebo-controlled trial in 60 patients diagnosed with MDD. The authors reported no significant improvements on the HAM-D, the Perceived Stress Scale (PSS-10), or the Symptom Checklist (SCL-90), but did see an improvement in two cognitive measures: the Attention and Perceptivity Test (APT) and California Verbal Learning Test (CVLT) (11). Finally, Chahwan et al. from Australia, also in 2019, examined the effects of a multispecies supplement containing *B. bifidum* W23, *B. lactis* W51, *B. lactis* W52, *L. acidophilus* W37, *L. brevis* W63, *L. casei* W56, *L. salivarius* W24, *Lactococcus lactis* W19, and *Lactococcus lactis* W58 in a sample of 71 depressed patients not currently taking any antidepressant medication and stratified into subgroups based on severity. While no significant improvements were reported on the BDI, the DASS, or the BAI, the authors did report a significant improvement in cognitive reactivity, measured using the Leiden Index of Depression Sensitivity-Revised (LEIDS-R) (12). This finding on cognitive reactivity to sad mood, a major risk factor for depression, is consistent with findings from a previous study in healthy controls (25). Taken together with the present findings, these studies provide a small foundation of research into this rapidly advancing field where further investigation is crucial.

The findings from the present study provide evidence for this combination of probiotics as a potential monotherapy for depression in treatment-naïve depressed patients. This line of research could have a major impact on many individuals seeking relief from depressive symptoms; Among other issues such as latency to produce a noticeable effect, antidepressant medication is often accompanied by intolerable side effects that cause a significant proportion of patients to discontinue their medication (26). These implications also have particular importance for populations for whom antidepressant medications are not recommended, such as those under 18 years of age, pregnant and breastfeeding women, the elderly, those with medical conditions taking medications that may present drug interactions, and those presenting with sub-clinical symptoms of depression. A daily probiotic supplement or a dietary increase in probiotic-rich foods could allow individuals to bypass stigma, latency, side effects, and barriers associated with antidepressant medication and still obtain relief from their depressive symptoms.

### Limitations

There are several limitations in the present study to acknowledge. First, the small sample size undermines the statistical interpretations and generalizability of the study. Second, the open-label design and lack of placebo makes the study liable to confirmation bias by both participants and study personnel. Third, the method of recruitment introduces an increased possibility of response bias; skewing the data toward a certain subset of patients who are more likely to respond to advertisements and participate in research, reducing the generalizability of the results. Fourth, the sample was disproportionately skewed toward young adult females. Demographic criteria for inclusion in the study were males and females 18–65 years, yet 90% of the sample was between 18 and 32 years and was 70% female. However, given the 2:1 female to male sex distribution of MDD, our sample is representative of the natural distribution of the disorder. The increased rate at which females seek treatment for mental health issues may also play a role in the distribution (27). Additionally, treatment-naïve patients experiencing moderate depression are naturally younger, explaining the relatively young average age of the sample. Fifth, our measurement of diet was inadequate in measuring how diet contributes to mental health and illness and thus was not included in any analyses. The brief diet questionnaire consisted of 5 questions assessing how often in the past month participants consumed red meat, sweetened beverages, refined sugar, fruits and vegetables, and multivitamins on a five-point Likert scale. A more comprehensive measure of diet, such as a detailed food frequency questionnaire, would provide more insight into how diet plays into depressive symptomatology and response to the probiotic supplement. Finally, there was no post-intervention follow-up period. A washout period following the intervention would have provided insight into how long clinical effects would be sustained, or whether prolonged use of probiotics would be required to maintain these effects.

### Future Directions

While the results from this study are promising, the existing limitations make it impossible to draw strong conclusions. To address this, the data from this pilot study was used to plan a double-blinded, randomized, placebo-controlled trial assessing the effects of the same probiotic supplement in participants diagnosed with MDD (28). In addition to clinical and polysomnographic measures, this expansion study features a neuroimaging data collection platform and extended molecular measures including genomics, proteomics, and metabolomics to explore potential biomarkers that will predict responsiveness to the probiotic. A larger sample size will allow us to stratify results by sex. This is crucial, given the sex differences observed in microbiome composition (29) which may influence gut-brain axis activity and response to the probiotic. We will also be able to more carefully examine common psychiatric comorbidities that may influence outcomes. Importantly, this study will also comprehensively examine potential mechanisms of action, such as how dietary patterns and nutritional status contribute to the relationship between probiotics and mental health, as well how microbial metabolites may be driving this relationship.

## Data Availability Statement

The raw data supporting the conclusions of this article will be made available by the authors, without undue reservation.

## Ethics Statement

The studies involving human participants were reviewed and approved by Queen's University Health Sciences and Affiliated Teaching Hospitals Research Ethics Board. The patients/participants provided their written informed consent to participate in this study.

## Author Contributions

CW and RM contributed to the design and implementation of the study. CW collected, analyzed, and interpreted the patient data and was a major contributor in writing the manuscript. All authors read and approved the final manuscript.

## Conflict of Interest

RM has received consulting and speaking honoraria from AbbVie, Allergan, Janssen, KYE, Lundbeck, Otsuka, and Sunovion, and research grants from CAN-BIND, CIHR, Janssen, Lallemand Health Solutions, Lundbeck, Nubiyota, OBI and OMHF. The funder did not contribute to the design of the study, implementation of the study, analyses of the data, or interpretation of the results. The remaining author declares that the research was conducted in the absence of any commercial or financial relationships that could be construed as a potential conflict of interest.

## Abbreviations

ANOVA: Analysis of variance
APT: Attention and Perceptivity Test
BDI: Beck Depression Inventory
CAN-BIND: Canadian Biomarker Integration Network in Depression
CFU: Colony forming units
CGI: Clinical Global Impression scale
CVLT: California Verbal Learning Test
DASS-42: Depression, Anxiety, and Stress Scale
DSM-IV: Diagnostic and Statistical Manual of Mental Disorders, 4th edition
ECT: Electroconvulsive Therapy
GAD-7: Generalized Anxiety Disorder 7-item scale
GAF: Global Assessment of Functioning
GI: Gastrointestinal
HAM-D: Hamilton Depression Rating Scale
LEIDS-R: Leiden Index of Depression Sensitivity-Revised
MADRS: Montgomery-Åsberg Depression Rating Scale
MDD: Major Depressive Disorder
MINI: Mini International Neuropsychiatric Interview
NHP: Natural Health Product
PSQI: Pittsburgh Sleep Quality Index
PSS-10: Perceived Stress Scale
QIDS-SR16: Quick Inventory of Depressive Symptomatology 16-item self-report
RCT: Randomized Control Trial
REM: Rapid Eye Movement
SCL-90: Symptom Checklist
SHAPS: Snaith Hamilton Pleasure Scale
SPSS: Statistical Package for Social Sciences
SSRI: Selective Serotonin Reuptake Inhibitor
STAI: State-Trait Anxiety Inventory.
